# 4-1BB Signaling Breaks the Tolerance of Maternal CD8^+^ T Cells That Are Reactive with Alloantigens

**DOI:** 10.1371/journal.pone.0045481

**Published:** 2012-09-21

**Authors:** Kwang H. Kim, Beom K. Choi, Jung D. Kim, Young H. Kim, Sun K. Lee, Jae H. Suh, Sang C. Lee, Sang W. Kang, Byoung S. Kwon

**Affiliations:** 1 Division of Cancer Biology, National Cancer Center, Goyang, Kyeonggi-do, Korea; 2 Departments of Obstetrics, Gynecology & Reproductive Sciences, Yale University School of Medicine, New Haven, Connecticut, United States of America; 3 Program for Immunotherapeutic Research, National Cancer Center, Goyang, Kyeonggi-do, Korea; 4 Department of Biological Sciences, University of Ulsan, Ulsan, Korea; 5 Department of Pathology, Ulsan University Hospital, Ulsan, Korea; 6 Department of Medicine, Tulane University Health Sciences Center, New Orleans, Louisianna, United States of America; Otto-von-Guericke University Magdeburg, Germany

## Abstract

4-1BB (CD137, TNFRSF9), a member of the activation-induced tumor necrosis factor receptor family, is a powerful T-cell costimulatory molecule. It generally enhances CD8^+^ T responses and even breaks the tolerance of CD8^+^ T cells in an antigen-specific manner. In the present study we found that it was expressed in the placentas of pregnant mice and that its expression coincided with that of the immunesuppressive enzyme indoleamine 2,3-dioxygenase (IDO). Therefore, we investigated whether 4-1BB signaling is involved in fetal rejection using agonistic anti-4-1BB mAb and 4-1BB-deficient mice. Treatment with agonistic anti-4-1BB mAb markedly increased the rate of rejection of allogeneic but not syngeneic fetuses, and this was primarily dependent on CD8^+^ T cells. Complement component 3 (C3) seemed to be the effector molecule because 4-1BB triggering resulting in accumulation of C3 in the placenta, and this accumulation was also reversed by anti-CD8 mAb treatment. These findings demonstrate that 4-1BB triggering breaks the tolerance of CD8^+^ T cells to alloantigens in the placenta. Moreover, triggering 4-1BB protected the pregnant mice from *Listeria monocytogenes* (LM) infection, but led to rejection of semi-allogeneic fetuses. Therefore, given the cross-recognition of alloantigen by pathogen-reactive CD8^+^ T cells, the true function of 4-1BB may be to reverse the hypo-responsiveness of pathogen-reactive CD8^+^ T cells in the placenta in cases of infection, even if that risks losing the fetus.

## Introduction

Since interactions between the maternal immune system and fetal tissues that express paternally inherited alloantigens should provoke immune responses against fetal tissues, successful pregnancy depends on mechanisms that suppress maternal immune activation by fetal alloantigens [1]. There is evidence that indoleamine 2,3-dioxygenase (IDO) is important in protecting the fetus from maternal rejection, either by suppressing activation of maternal decidual T lymphocytes [2] or by acting as an antioxidant enzyme [3].

4-1BB (CD137, TNFRSF9), a member of the tumor necrosis factor (TNF) receptor superfamily, functions as an inducible costimulatory molecule for T cells [4], [5], [6]. Although 4-1BB signaling has dual roles in modulating immune responses [7], 4-1BB triggering *in vivo* generally enhances immune responses against tumors or viruses by preferentially stimulating CD8^+^ T responses [4], [8].

Although there have been many studies of the expression of costimulatory molecules in placental tissue and immune cells [9], [10], few studies have focused on the functional aspects of costimulatory molecules in the placenta and in placental immune cells *in vivo* [11]. It has been reported that diverse members of the TNF superfamily are expressed in placental tissue [12] and 4-1BB is also expressed in the pregnant mouse uterus [13]. We found that 4-1BB was co-induced with IDO from about 8 days post-coitus. Since IDO plays crucial roles in maintaining maternal immunological tolerance during pregnancy [2], the co-induction of 4-1BB with IDO raised a question whether 4-1BB was increased to suppress or enhance the immune responses in the pregnant mice.

Therefore, we examined the possible roles of 4-1BB signaling in the placenta using agonistic anti-4-1BB mAb, blocking anti-4-1BB ligand mAb, and 4-1BB-deficient mice, and found that 4-1BB triggering resulted in rejection of semi-allogeneic fetuses, but was required to protect the pregnant mice from infections.

## Results

### 4-1BB Expression in the Placenta during Pregnancy

**Figure 1:**
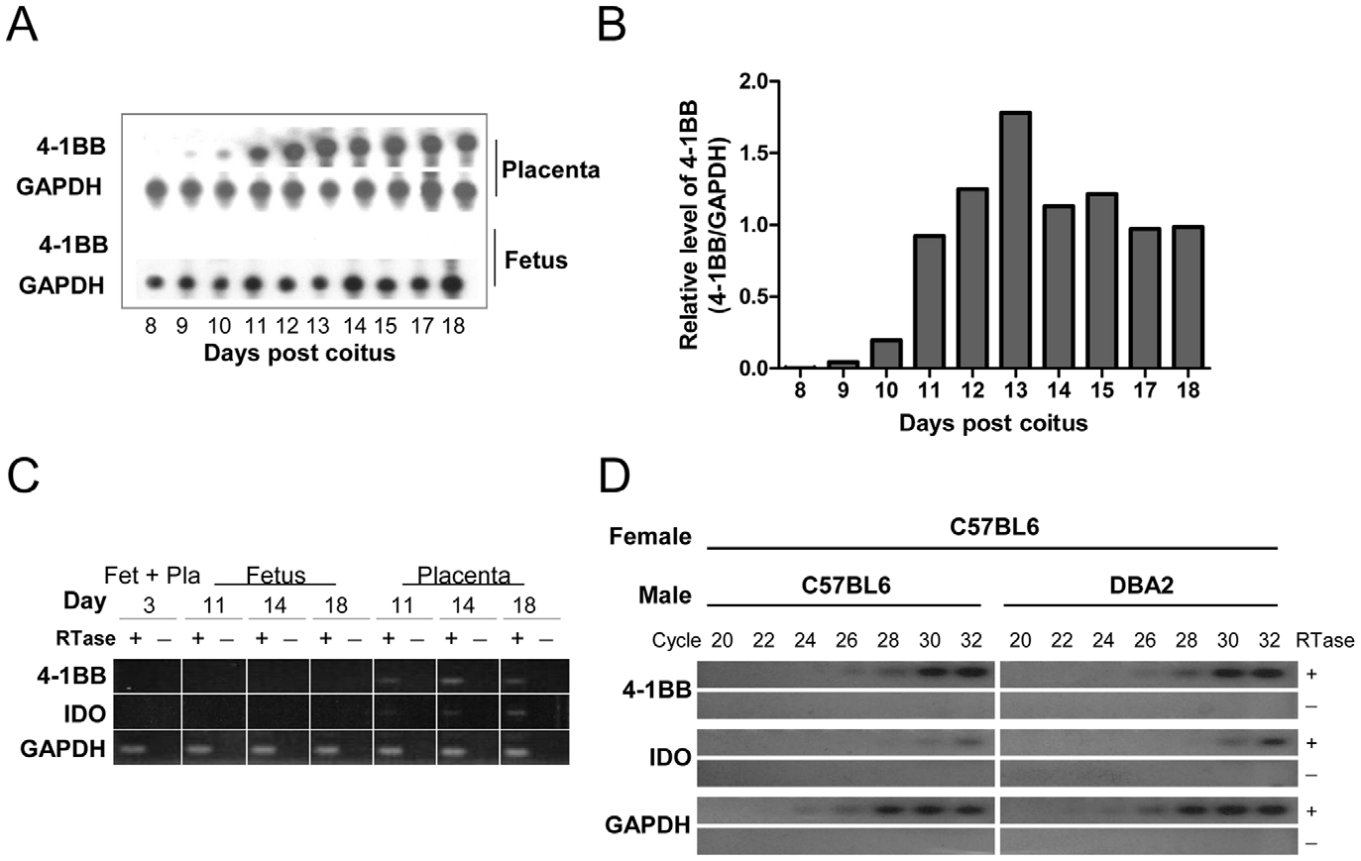
4-1BB induction in the placenta during pregnancy. (A) Outbred ICR mice were time-mated, and placenta and fetal tissues were collected at the indicated days post coitus (dpc) for RNA extraction. An RNase protection assay (RPA) was performed to determine the mRNA levels of 4-1BB and GAPDH. (B) Expression levels of 4-1BB and GAPDH were measured by densitometer and the relative expression levels of 4-1BB were calculated by dividing the densitometry values for 4-1BB by those for GAPDH. (C) First-strand cDNA synthesis was synthesized using fetal and/or placental tissue at the indicated dpc with or without reverse transcriptase (RTase), and 4-1BB, IDO, and GAPDH transcripts were amplified by 30 cycles of PCR. (D) C57BL/6 female mice were time-mated with C57BL/6 or DBA/2 males, and total RNAs were isolated from the placental tissues at 15 dpc. First-strand cDNAs were synthesized with and without RTase, and 20, 22, 24, 28, 30, 32 PCR cycles were performed.

To examine the role of 4-1BB in placental and fetal development, outbred ICR mice were time-mated, and tissues were prepared from embryo, and placenta, on the indicated days. Total RNA was isolated from the tissues, and ribonuclease protection assays (RPA) were performed to measure expression of 4-1BB, along with GAPDH as a control. 4-1BB mRNA was first detected in the placenta at eight days post coitus (dpc) and reached a plateau at 11 dpc (Figure 1A and 1B). It was not detected in the fetus from 8 to18 dpc (Figure 1A).

We compared placental expression of 4-1BB with that of indoleamine 2,3-dioxygenase (IDO) in the placenta by PCR. 4-1BB and IDO expression coincided in the placenta, and neither was detected in the fetus (Figure 1C). Since the mice used in these experiments were outbred, we tested whether the placental 4-1BB induction was due to the semi-allogeneic nature of the fetuses by mating C57BL/6 female mice with C57BL/6 or DBA/2 male mice (Figure 1D). We found that 4-1BB and IDO were expressed to similar extents in the mice carrying syngeneic and allogeneic fetuses, indicating that fetal alloantigen are not responsible for the placental expression of 4-1BB.

### 4-1BB Triggering in vivo Leads to Rejection of Allogeneic Fetuses

We set up matings between C57BL/6 females and C57BL/6 or DBA/2 males to examine the effect of 4-1BB signaling on syngeneic *versus* allogeneic fetuses by treating them with agonistic anti-4-1BB or blocking anti-4-1BBL mAb. We checked C57BL/6 females for vaginal plugs, treated them with 200 µg of antibodies at 8 and 12 dpc and measured pregnancy outcomes by counting total numbers of pups and numbers of pups per female. Mean pups per pregnant C57BL/6 females carrying syngeneic and allogeneic fetuses were quite similar –7.1±1.22 and 7.3±0.96, respectively (Table 1). Blocking the 4-1BB/4-1BBL interaction by treating with anti-4-1BBL mAb did not alter the delivery of allogeneic litters (6.7 pups on average), and was not tested on mice carrying syngeneic fetuses. However, C57BL/6 mice mated with DBA/2 mice and treated with agonistic anti-4-1BB mAb delivered only 2.7 pups on average, while the same treatment had no effect on 4-1BB-deficient mice carrying 4-1BB-positive allogeneic fetuses (7.7 pups on average), and slightly but not significantly reduced the delivery of syngeneic fetuses (5.9 pups on average) (Table 1). These observations suggest that 4-1BB triggering only induces the rejection of allogeneic fetuses.

**Table 1:** Effect of the 4-1BB-4/1BBL interaction on allogeneic and syngeneic concepti.

Treatmentperiod	Female	Male	Treatment	Numberpregnant	Number of neonates	Pups per female(average)
E8–12	C57BL/6	C57BL/6	Rat IgG	16	109	6.8±1.27
			Anti-4-1BBL	5	36	7.2±0.84
			Anti-4-1BB	10	59	5.9±1.37
	C57BL/6	DBA/2	Rat IgG	9	66	7.3±1.00
			Anti-4-1BBL	7	47	6.7±1.25
			Anti-4-1BB	16	43	2.7±1.74***
	4-1BB-deficient C57BL/6		Rat IgG	8	62	7.8±1.03
			Anti-4-1BB	9	69	7.7±1.00

Wild-type or 4-1BB-deficient female mice in a C57BL/6 background (8 weeks-old) were mated with syngeneic C57BL/6 or allogeneic DBA/2 males. Control rat IgG, blocking anti-4-1BBL mAb (TKS-1) or agonistic anti-4-1BB (3E1) was injected i.p. into the mice at 200 µg per mouse 8, 10, and 12 days after mating. Data are means ± SD. *P* values are calculated relative to the rat IgG-treated controls: ***P<0.0001.

**Figure 2:**
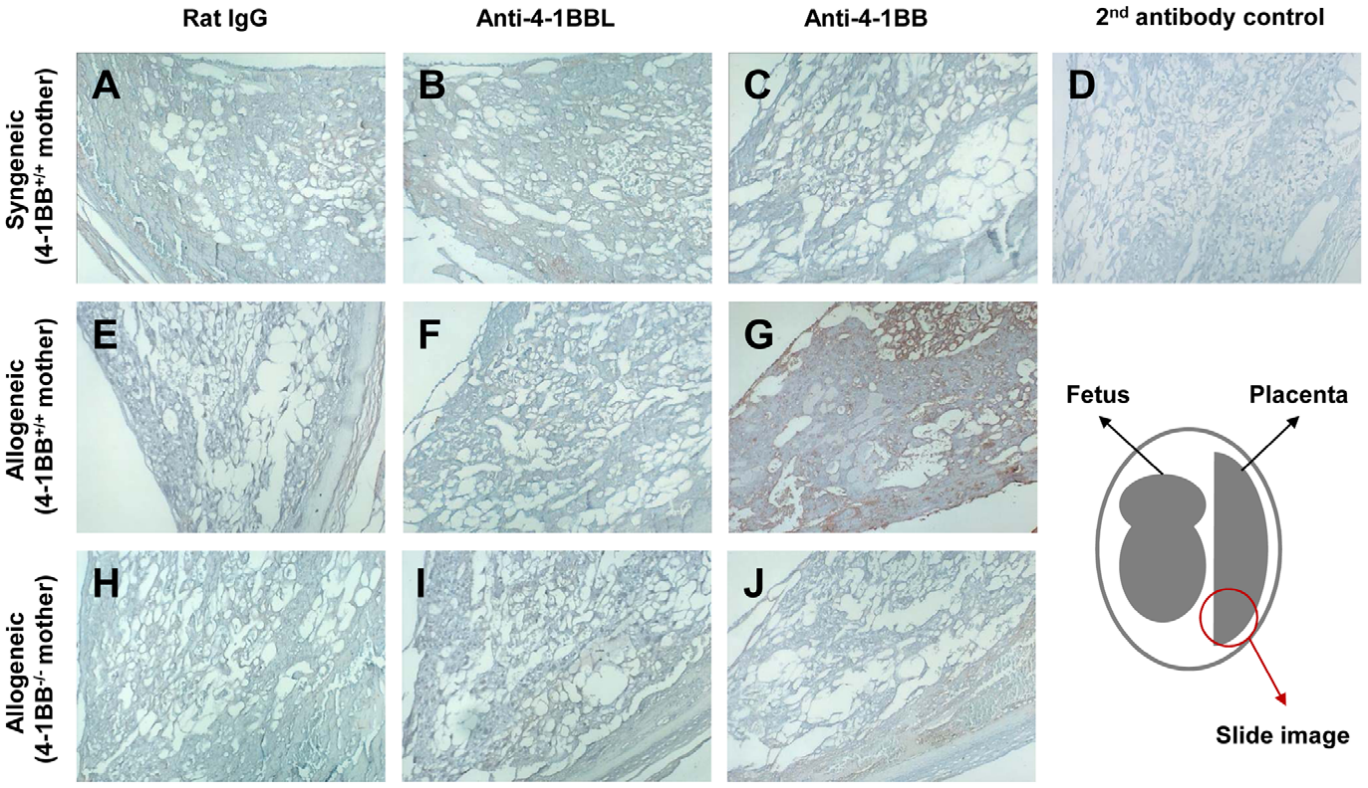
4-1BB triggering leads to rejection of allogeneic fetuses. C57BL/6 females were time-mated with C57BL/6 (A-D) or DBA/2 male mice (E-G), while 4-1BB KO C57BL/6 females were time-mated with DBA/2 male mice (H-J) (n  = 5 per group). Female mice with vaginal plugs were separated into three groups and injected i.p. with 200 µg of rat IgG (A, E, H), blocking anti-4-1BBL (B, E, F) or agonistic anti-4-1BB mAb (C, G, J), at 8, 10, and 12 dpc. At 15 dpc, two or three pregnant mice were randomly selected from each group and three gravid uteri were collected from each pregnant mouse by avoiding dead fetuses. The gravid uteri were used to prepare paraffin blocks. Paraffin sections were stained with primary rabbit anti-human C3, followed by secondary HRP-conjugated anti-rabbit IgG. Bound antibody was detected with a Vector DAB substrate kit, and the sections were counterstained with hematoxylin. Paraffin secions were stained with secondary HRP-conjugated anti-rabbit IgG alone as a negative control (D). Original magnification ×20. Data are representative of two independent experiments.

Since complement has been proposed as a common effector molecule causing fetal injury in response to different triggers [14], [15], we performed immunohistological staining to see whether complement accumulated in the placenta following treatment with anti-4-1BB mAb. We found extensive deposition of complement C3 over the whole placenta, including the deciduas. Complement C3 deposition in the placenta was barely detectable in mice carrying syngeneic fetuses (Figure 2A-C) or in rat IgG- or anti-4-1BBL mAb-treated mice carrying allogeneic fetuses (Figure 2E, F). However, we found extensive deposition of C3 over the whole placenta, including the decidua, in pregnant mice that were mated with DBA/2 mice and received agonistic anti-4-1BB mAb (Figure 2G). C3 deposition was not detected under any conditions in the placentas of 4-1BB-deficient mice carrying allogeneic fetuses (Figure 2H-J). These results again demonstrate that 4-1BB triggering *in vivo* selectively induces the rejection of allogeneic fetuses.

### 4-1BB-mediated Rejection of Allogeneic Fetuses is CD8^+^ T Cell-dependent

Since 4-1BB signaling preferentially activates T cells [8], we suspected that these cells might be the ones responsible for the 4-1BB-mediated fetal rejection. To evaluate the role of maternal CD4^+^ T and CD8^+^ T cells, pregnant mice carrying syngeneic or allogeneic fetuses were treated with depleting anti-CD4 (GK1.5) or anti-CD8 (2.43) mAb at 8, 10, and 12 dpc. Again, the mean number of pups per female carrying syngeneic fetuses was not significantly changed by 4-1BB triggering, or by depleting CD4^+^ T or CD8^+^ T cells (Table 2). However, 4-1BB triggering induced fetal rejection of allogeneic fetuses and this was completely prevented by depleting CD8^+^ T cells, but not by depleting CD4^+^ T cells.

**Table 2:** CD8^+^ T cells mediate allogeneic fetal rejection by agonistic anti-4-1BB mAb.

Treatmentperiod	Female	Male	Treatment	Numberpregnant	Number ofneonates	Pups per female(average)
E8–12	C57BL/6	C57BL/6	Rat IgG	5	40	8.0±1.22
			Anti-4-1BB	5	36	7.2±0.83
			Anti-4-1BB + anti-CD4	5	41	8.2±1.30
			Anti-4-1BB + anti-CD8	5	44	8.8±0.84
	C57BL/6	DBA/2	Rat IgG	5	45	9.0±0.70
			Anti-4-1BB	5	7	1.4±1.34
			Anti-4-1BB + anti-CD4	5	10	2.0±1.58
			Anti-4-1BB + anti-CD8	5	39	7.8±1.83**

C57BL/6 female mice were mated with syngeneic C57BL/6 male or allogeneic DBA/2 male mice. 200μg agonistic anti-4-1BB (3E1) mAb or rat IgG as a control was injected i.p. into the mice 8, 10, 12 days post coitus. Some of the anti-4-1BB mAb-treated mice also received 400 µg per mouse of depleting anti-CD4 (GK1.5) or anti-CD8 (2.43) mAb on days 8 and 12. The values shown represent the means ± SDs of two independent experiments. *P* values were calculated relative to the mice receiving only anti-4-1BB mAb: **P<0.01.

**Figure 3:**
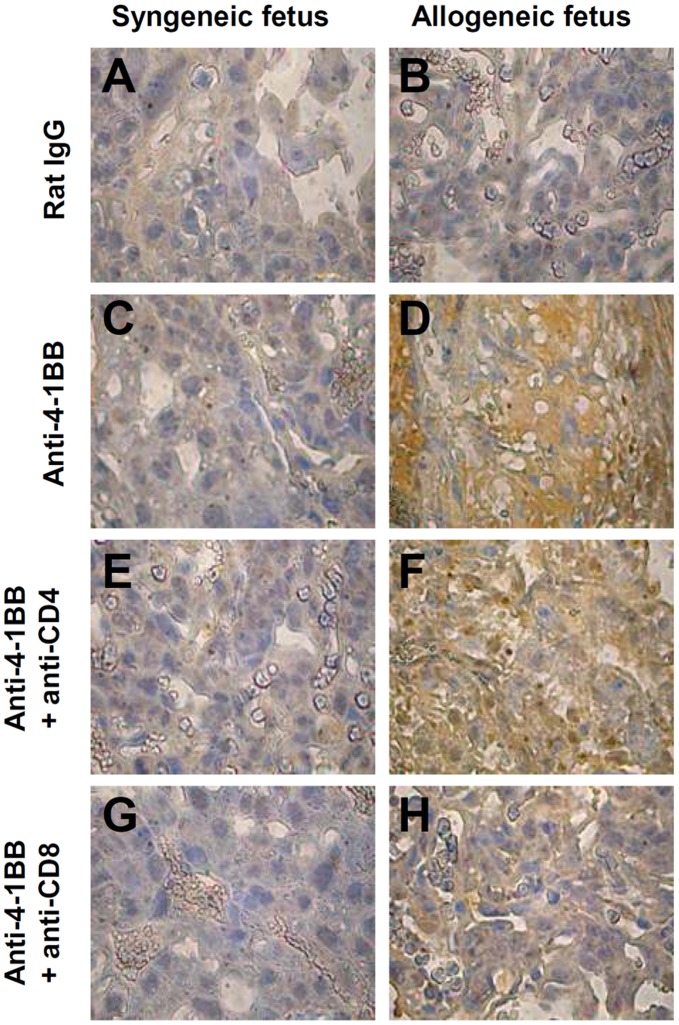
4-1BB triggering induces allogeneic fetal rejection through CD8^+^ T cells. C57BL/6 female mice were time-mated with C57BL/6 (A, C, E, G; n  = 25 female) or DBA/2 male mice (B, D, F, H; n  = 25 female). Mice with vaginal plugs were selected and separated into groups (n  = 3–4) and injected i.p. with rat IgG (A, B) or anti-4-1BB mAb (C, D, E, F, G, H) as described above. The mice received 400 µg of anti-CD4 (E, F) or anti-CD8 (G, H) mAb along with anti-4-1BB mAb at 8 and 12 dpc. Gravid uteri were collected from 2 - 3 pregnant mice in each group at 15 dpc and C3 deposition was detected with anti-human C3 Ab and HRP-conjugated secondary Ab. Bound antibody was detected with a Vector DAB substrate kit and sections were counterstained with hematoxylin. Original magnification ×80. Data are representative of two (A, C, E and G) or three (B, D, F and H) independent experiments.

To show that depletion of CD8^+^ T cells reduces complement deposition, placental tissues were collected from each group and stained with anti-C3 antibody. As anticipated the C3 deposition in 4-1BB-treated mice carrying allogeneic fetuses (Figure 3D), was markedly reduced by depleting CD8^+^ T cells (Figure 3H), but not by depleting CD4^+^ T cells (Figure 3F). C3 deposition was not detectable in any of the mice carrying syngeneic fetuses (Figure 3A, 3C, 3E, and 3G). These results indicate that maternal CD8^+^ T cells play crucial roles in the 4-1BB-mediated rejection of allogeneic fetuses.

### 4-1BB Triggering Protects Pregnant Mice from Listeria Monocytogenes (LM) Infection

**Figure 4:**
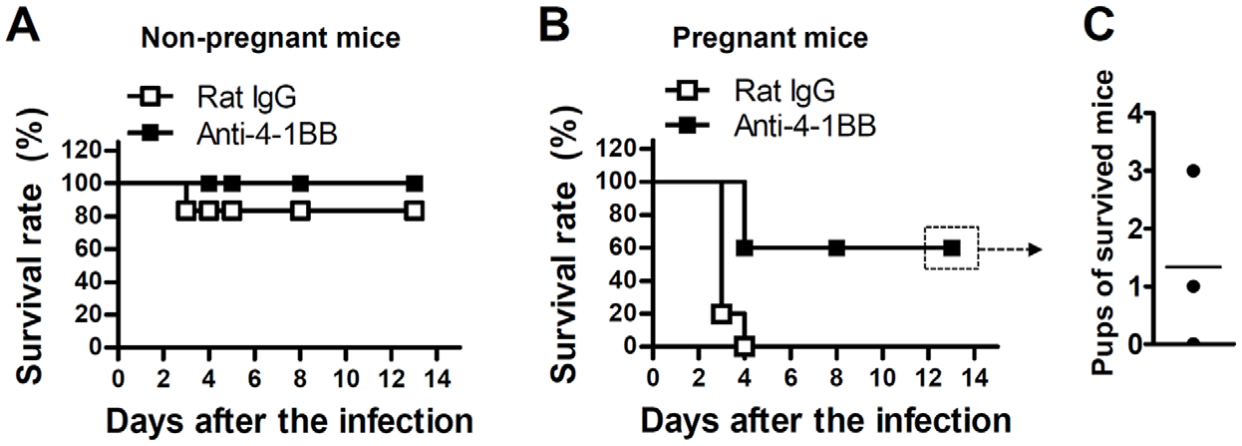
Anti-m4-1BB protects pregnant mice from LM infection. (A) Non-pregnant or (B) pregnant C57BL/6 (at 8 dpc) mice were infected i.v. with 5×10^3 ^CF of LM and concurrently injected i.p. with anti-4-1BB mAb, or rat IgG as a control (n  = 5). Pup numbers were counted 13 days after infection of the 4-1BB-injected pregnant mice (C). Experiments were repeated two (A) or three (B and C) times with similar results.

The reduced immuno-responsiveness associated with pregnancy is required to sustain pregnancy but increases susceptibility to infection [16], [17]. Therefore, we hypothesized that 4-1BB might be increased in the placenta to reverse the maternal tolerance when an infection occurs that could cause maternal death. To test this idea, we mated female C57BL/6 mice with male DAB/2 mice, infected them i.v. with 5×10^3^ CFU of *Listeria monocytogenes* (LM) on dpc 8, and administered them concurrently with anti-4-1BB mAb or rat IgG on dpc 8 and 12. Non-pregnant mice were also infected with 5×10^3^ CFU of LM on dpc 8, and concurrently given anti-4-1BB mAb or rat IgG. In the non-pregnant IgG-treated mice, LM infection resulted in 20% mortality, and there was no death in the anti-4-1BB treated mice (Figure 4A). The pregnant mice were more susceptible to the infection than the non-pregnant mice as previously reported [16], and all of them died within 3–4 days of infection (Figure 4B). Those pregnant mice that received anti-4-1BB mAb were more resistant, and more than half of them survived the infection (Figure 4B). However they gave birth to very few pups or no birth (Figure 4C).

These results indicate that 4-1BB triggering protects pregnant mice from LM infection, but at the same time leads to the rejection of semi-allogeneic fetuses.

## Discussion

Here we found that 4-1BB and IDO increased simultaneously in the placenta during pregnancy (Figure 1). Since 4-1BB and IDO have opposite effects on immune responses [18], we attempted to understand the 4-1BB increase. We found that triggering of 4-1BB signaling *in vivo* reduced the delivery of semi-allogeneic pups, while the endogenous level of 4-1BB signaling did not have this effect (Table 1). The loss of allogeneic fetuses as a result of 4-1BB triggering was primarily mediated by CD8^+^ T cells (Table 2 and Figure 3). However, since triggering 4-1BB protected the pregnant mice from LM infection (Figure 4), we infer that 4-1BB expression increases in the placenta during pregnancy to protect the pregnant mother from emergencies.

It is well-known that 4-1BB signaling preferentially induces the proliferation and differentiation of CD8^+^ T cells [19]. However, there is increasing evidence that it exerts the regulatory effect by inducing IDO in antigen-presenting cells [18], [20], [21], [22]. Therefore, when we first observed the co-induction of 4-1BB and IDO in the placenta during pregnancy (Figure 1), it was obscure whether the 4-1BB was contributing to the induction and maintenance of IDO expression in the placenta or accumulated to antagonize the IDO increase. Since we found that inhibiting the 4-1BB/4-1BBL interaction had only a marginal effect on the delivery of allogeneic pups, whereas 4-1BB triggering markedly reduced their delivery (Table 1), we concluded that the increased 4-1BB expression in the placenta was acting to break maternal tolerance in the placenta.

Although the matter is still controversial, evidence from studies of murine and human pregnancies indicates that maternal Th2-type immunity is associated with successful pregnancy, while Th1-type immune reactivity is associated with miscarriages [14], [23]. We found C3 deposition in the placentas of mice carrying allogeneic fetuses following treatment with anti-4-1BB mAb (Figure 2G). 4-1BB triggering typically polarizes Th1 responses by enhancing CD8^+^ T cell responses and suppressing humoral responses [24], [25], [26]. The observations that (1) IDO protects the fetus by suppressing T cell-driven complement activation [2], (2) hyper-stimulation with 4-1BB causes fetal rejection via CD8^+^ T cells (Table 2), and (3) spleen size and numbers of splenocytes increase in mice carrying allogeneic fetuses in response to anti-4-1BB mAb (Figure S1), suggest that 4-1BB-triggering of T cells reactive with alloantigen may be somehow linked to complement activation. The fact that depletion of CD8^+^ T cells abolished anti-4-1BB-mediated fetal loss and complement deposition in the placenta, while depletion of CD4^+^ T cells did not (Table 2 and Figure 3F, H), is inconsistent with a previous report that CD8^+^ T cells prevented spontaneous abortion in DBA/2-mated CBA/J mice [27], but does agree with the observation that the Th1 cytokine profile induces miscarriages in humans and mice [28].

There are at least two forms of 4-1BB resulting from alternative splicing of the 4-1BB transcript: a functional type I transmembrane protein and a secreted soluble protein [29], [30]. The soluble 4-1BB is thought to be a decoy receptor that may have immune-suppressive activity [30]. It was shown recently that two forms of 4-1BB are expressed in the mouse uterus and conceptus during implantation [13]. Eckstrum et al. speculated that the soluble form might be involved in suppression of the maternal immune system [13]. As the membrane form increases during pregnancy, the soluble form may also need to increase to prevent activation of 4-1BB-expressing immune cells.

Nevertheless, the experiments presented above clearly demonstrate that 4-1BB triggering with agonistic anti-4-1BB mAb leads to allogeneic fetal loss by breaking the tolerance of maternal CD8^+^ T cells, which indicates that agonistic anti-4-1BB mAb may overcome the suppressive function of soluble 4-1BB. Given the magnitude of 4-1BB signaling needed to induce fetal rejection, similar phenomena may only occur in cases of severe inflammation such as acute virus infection. Due to the hypo-responsiveness of adaptive immunity during pregnancy [28], the possibility that such infections cannot be controlled is increased [17]. In that kind of crisis, 4-1BB costimulation seems to awaken maternal CD8^+^ T cells from their hypo-responsiveness in order to eradicate the pathogen despite the resulting risk of rejecting an allogeneic fetus due to cross-recognition of alloantigens and conventional antigens by the CD8^+^ T cells [31].

Taken together, our results provide the first evidence that 4-1BB signaling plays a role in activating maternal CD8^+^ T cells from their hypo-responsiveness that are reactive with allogeneic fetal tissue and probably with conventional antigens.

## Materials and Methods

### Mice

All animal studies were approved by the Institutional Animal Care and Use Committee (IACUC) review board of National Cancer Center (NCC-09-070) and conducted under the guidelines of the National Cancer Center IACUC. The C57BL/6, DBA/2, and ICR mice used in these studies were obtained from the Jackson Laboratory, (Maine, USA) and homozygous 4-1BB-deficient mice [32] were backcrossed for at least 12 generations with wild-type C57BL/6 mice, also from the Jackson Laboratory. All mice were kept in specific pathogen-free conditions in the animal facility of the NCC (National Cancer Center, Korea).

### Reagents

Agonistic anti-4-1BB mAb (3E1) was kindly provided by Dr. R. Mitter (Emory University, USA) and blocking anti-4-1BBL mAb (TKS-1) by Dr. K. Okumura (Juntendo University, Japan). Rabbit anti-human C3d complement, and polyclonal goat anti-rabbit immunoglobulin/HRP conjugates, were purchased from Dako. *Listeria monocytogenes* (LM; strain 10403S, ATCC) was passaged in mice to maintain virulence, and cultured in Brain Heart Infusion (BHI) broth (Difco Laboratories).

### Timed Matings

Eight-week-old female C57BL/6 and 4-1BB-deficient C57BL/6 mice were mated with nine-week-old male DAB/2 or C57BL/6 mice. Females were inspected daily for vaginal plugs and those with plugs were removed from breeding cages at 0.5 days post coitus (dpc).

### RNA Analysis

Total RNA was prepared from homogenized placentas or fetal tissue using Trizol reagent (Invitrogen). RT-PCR was performed as specified in the RT-PCR kit (Invitrogen). All reactions were run in duplicate using cDNA templates synthesized from 1 µg samaples of RNA. PCR was performed in 20 µl reaction mixtures containing cDNA and 0.5 µM of the following primer pairs: for 4-1BB (sense, 5′- CGA AAC CGA GAA GCA CTA GG-3′ and anti-sense, 5′- CTC AGG CAT CAG GAG TGT CA - 3′), for mGAPDH (sense, 5′- GAA CGG GAA GCT TGT CAT CAA - 3′ and anti-sense, 5′- CTA AGC AGT TGG TGT GCA G - 3′), and for mIDO (sense, 5′-GCC AAG TGG GGG GTC AGT GGA GTA GAC A-3′ and anti-sense, 5′- CCC TGA TAG AAG TGG AGC TTG CTA CAC TA - 3′).

### RNase Protection Assays (RPA)

A 4-1BB probe fragment including the 5′ untranslated regions (UTRs) of exon 1 and exon 2 (223 bp) and a GAPDH probe fragment (97 bp) were amplified with the following primers: 4-1BB sense, 5′- TCC ATG AAC TGC TGA GTG G - 3′; 4-1BB anti-sense, 5′ - GGC GAA ATG TCA CAT GCA - 3′; GAPDH sense, 5′- CTC AAG ATT GTC AGC AAT GCA TC-3′; GAPDH anti-sense, 5′- TCA TGA GCC CTT CCA CAA TG-3′. The amplified PCR products were cloned into pGEM-T Easy vector (Promega) and clones with the SP6 promoter orientation were selected. To prepare probe anti-sense, Nde I-digested linearized fragments were used for *in vitro* transcription by T7 RNA polymerase. The RNase protection assay was performed according to the Pharmingen standard protocol using the Riboquant *in vitro* Transcription and RPA kits (BD PharMingen). Briefly, 5 µg of total RNA was hybridized overnight with [α-^32^P]UTP (Amersham Biosciences, Piscataway, NJ)-labeled probes at 56°C. Unhybridized ssRNA was digested by RNase treatment, and the dsRNA was purified by phenol/chloroform extraction and ethanol precipitation. The RNA pellet was resuspended in 5 µl of RNA sample loading buffer. The samples were fractionated by electrophoresis on a 6% polyacrylamide/7 M urea gel, dried, and exposed to X-ray film (Agfa, Ridgefield Park, NJ) for autoradiographic analysis.

Densitometry values of 4-1BB and GAPDH were calculated using the ‘Image J’ program, and the relative expression of 4-1BB was calculated by dividing the densitometry values for 4-1BB by the values for GAPDH.

### Immunohistochemistry

To detect C3 complement, 2- 3 pregnant mice were randomly selected from each group of mice (5 females per group) at 15 dpc and three gravid uteri were further obtained from the selected mice by avoiding dead fetuses. The selected uteri were fixed overnight in 10% neutral-buffed formalin, embedded in paraffin. Five randomly selected 5 mm sections were cut from each uterus tissue and used to stain the C3 complement. Endogenous peroxidase was quenched with 10% H_2_O_2_ in distilled water. The sections were first incubated in normal rabbit serum to block nonspecific binding (Vector), then with primary C3 antibody, followed by secondary anti-goat IgG or anti-rabbit IgG/HRP. Bound antibody was detected with 3, 3-diaminobenzidine tetrahydrochloride (DAB) (Vector) for 2 min and rinses in distilled water. Sections were counterstained with hematoxylin.

### Listeria Monocytogenese (LM) Infection

Female C57BL/6 mice were mated with male DAB/2 mice and inspected for vaginal plugs as described above. Mice with plugs were infected intravenously (i.v.) with 5×10^3^ colony forming unit (CFU) of LM at 8 dpc and i.p. injected concurrently with anti-4-1BB mAb or rat IgG as a control. The mice were monitored daily for survival.

### Statistical Analysis

All data were analyzed with the statistical program, Prism 4.0 GraphPad (San Diego, CA). Student’s t-test was used to determine the statistical significance of differences between groups.

## Supporting Information

Figure S1 **Impacts of 4-1BB triggering on splenocytes of the mice carrying syngeneic or allogeneic fetus.** C57BL/6 female mice were time-mated with C57BL/6 (b, e) or DBA/2 (c, f) male mice. The mice with vaginal plug and non-pregnant C57BL/6 female mice were further injected i.p. with rat IgG or anti-4-1BB mAb at 8, 10, and 12 dpc as described above. The mice were sacrificed on 15 dpc and spleens were photographed (A) and total splenocytes were counted (B).
(TIF)

## References

[1] Trowsdale J, Betz AG (2006) Mother’s little helpers: mechanisms of maternal-fetal tolerance. Nat Immunol 7: 241–246. 16482172 10.1038/ni1317

[2] Mellor AL, Sivakumar J, Chandler P, Smith K, Molina H, et al (2001) Prevention of T cell-driven complement activation and inflammation by tryptophan catabolism during pregnancy. Nat Immunol 2: 64–68. 11135580 10.1038/83183

[3] Hayaishi O, Hirata F, Ohnishi T, Henry JP, Rosenthal I, et al (1977) Indoleamine 2,3-dioxygenase: incorporation of 18O2− and 18O2 into the reaction products. J Biol Che 252: 3548–3550. 193836

[4] Melero I, Shuford WW, Newby SA, Aruffo A, Ledbetter JA, et al (1997) Monoclonal antibodies against the 4-1BB T-cell activation molecule eradicate established tumors. Nat Med 3: 682–685. 9176498 10.1038/nm0697-682

[5] Kwon BS, Weissman SM (1989) cDNA sequences of two inducible T-cell genes. Proc Natl Acad Sci U S A 86: 1963–1967. 2784565 10.1073/pnas.86.6.1963 PMC286825

[6] Pollok KE, Kim YJ, Zhou Z, Hurtado J, Kim KK, et al (1993) Inducible T cell antigen 4-1BB. Analysis of expression and function. J Immunol 150: 771–781. 7678621

[7] Vinay DS, Cha K, Kwon BS (2006) Dual immunoregulatory pathways of 4-1BB signaling. J Mol Med 84: 726–736. 16924475 10.1007/s00109-006-0072-2

[8] Halstead ES, Mueller YM, Altman JD, Katsikis PD (2002) In vivo stimulation of CD137 broadens primary antiviral CD8^+^ T cell responses. Nat Immunol 3: 536–541. 12021777 10.1038/ni798

[9] Larsen CP, Knechtle SJ, Adams A, Pearson T, Kirk AD (2006) A New Look at Blockade of T-cell Costimulation: A Therapeutic Strategy for Long-term Maintenance of Immunosuppression. Am J Transplant 6: 876–883. 16611323 10.1111/j.1600-6143.2006.01259.x

[10] Yoshimatsu J, Matsumoto H, Narahara H (2006) Co-stimulatory molecule OX40 ligand in early human pregnancy. Int J Gynaecol Obstet 93: 240–241. 16626714 10.1016/j.ijgo.2006.02.020

[11] Vacchio MS, Hodes RJ (2007) CD28 costimulation is required for in vivo induction of peripheral tolerance in CD8^+^ T cells. J Exp Med 26: 19–26. 10.1084/jem.20021429 PMC2193800 12515810

[12] Phillips TA, Ni J, Hunt JS (2001) Death-Inducing Tumour Necrosis Factor (TNF) superfamily ligands and Receptors are Transcribed in human placentae, cytotrophoblasts, Placental Macrophages and Placental Cell Lines. Placenta 22: 663–672. 11597186 10.1053/plac.2001.0703

[13] Eckstrum K, Bany BM (2011) Tumor necrosis factor receptor subfamily 9 (Tnfrsf9) gene is expressed in distinct cell populations in mouse uterus and conceptus during implantation period of pregnancy. Cell Tissue Res 344: 567–576. 21560035 10.1007/s00441-011-1171-0 PMC3104000

[14] Girardi G, Salmon JB (2003) The role of complement in pregnancy and fetal loss. Autoimmunity 36: 19–26. 12765467 10.1080/0891693031000067322

[15] Xu C, Mao D, Holers VM, Palanca B, Cheng AM, et al (2000) A critical role for murine complement regulator crry in fetomaternal tolerance. Science 287: 498–501. 10642554 10.1126/science.287.5452.498

[16] Luft BJ, Remington JS (1982) Effect of pregnancy on resistance to Listeria monocytogenes and Toxoplasma gondii infections in mice. Infect Immun 38: 1164–1171. 6818146 10.1128/iai.38.3.1164-1171.1982 PMC347871

[17] Constantin CM, Masopust D, Gourley T, Grayson J, Strickland OL, et al (2007) Normal establishment of virus-specific memory CD8 T cell pool following primary infection during pregnancy. J Immunol 179: 4383–4389. 17878333 10.4049/jimmunol.179.7.4383

[18] Seo SK, Choi JH, Kim YH, Kang WJ, Park HY, et al (2004) 4-1BB-mediated immunotherapy of rheumatoid arthritis. Nat Med 10: 1088–1094. 15448685 10.1038/nm1107

[19] Shuford WW, Klussman K, Tritchler DD, Loo DT, Chalupny J, et al (2007) 4-1BB costimulation signals preferentially induce CD8^+^ T cell proliferation and lead to the amplification in vivo of cytotoxic T cell responses. J Exp Med 26: 47–55. 10.1084/jem.186.1.47 PMC2198949 9206996

[20] Sun Y, Lin X, Chen HM, Wu Q, Subudhi SK, et al (2002) Administration of agonistic anti-4-1BB monoclonal antibody leads to the amelioration of experimental autoimmune encephalomyelitis. J Immunol 168: 1457–1465. 11801689 10.4049/jimmunol.168.3.1457

[21] Choi BK, Asai T, Vinay DS, Kim YH, Kwon BS (2006) 4-1BB-mediated amelioration of experimental autoimmune uveoretinitis is caused by indoleamine 2, 3-dioxygenase-dependent mechanisms. Cytokine 34: 233–242. 16899371 10.1016/j.cyto.2006.04.008

[22] Myers L, Takahashi C, Mittler RS, Rossi RJ, Vella AT (2003) Effector CD8 T cells possess suppressor function after 4-1BB and Toll-like receptor triggering. Proc Natl Acad Sci U S A 100: 5348–5353. 12695569 10.1073/pnas.0837611100 PMC154348

[23] Raghupathy R (2001) Pregnancy: success and failure within the Th1/Th2/Th3 paradigm. Semin Immunol. 13: 219–227. 10.1006/smim.2001.0316 11437629

[24] Mittler RS, Bailey TS, Klussman K, Trailsmith MD, Hoffmann MK (1999) Anti-4-1BB monoclonal antibodies abrogate T cell-dependent humoral immune responses in vivo through the induction of helper T cell anergy. J Exp Med 190: 1535–1540. 10562327 10.1084/jem.190.10.1535 PMC2195697

[25] Polte T, Foell J, Werner C, Hoymann HG, Braun A, et al (2006) CD137-mediated immunotherapy for allergic asthma. J Clin Invest 116: 1025–1036. 16528411 10.1172/JCI23792 PMC1395480

[26] Hellstrom KE, Hellstrom I (2003) Therapeutic vaccination with tumor cells that engage CD137. Mol Med 81: 71–86. 10.1007/s00109-002-0413-8 12601523

[27] Joachim RA, Hildebrandt M, Oder J, Klapp BF, Arck PC (2001) Murine stress-triggered abortion is mediated by increase of CD8+ TNF-alpha+ decidual cells via substance P. Am J Reprod Immunol. 45: 303–309. 10.1111/j.8755-8920.2001.450506.x 11432405

[28] Makhseed M, Raghupathy R, Azizieh F, Omu A, Al-Shamali E, et al (2001) Th1 and Th2 cytokine profiles in recurrent aborters with successful pregnancy and with subsequent abortions. Hum Reprod 16: 2219–2226. 11574519 10.1093/humrep/16.10.2219

[29] Schwarz H (2006) Significance of reverse signal transduction for the biology of the CD137 receptor/lignad system. In: Chen L, editor. CD137 Pathway. Springer: New York. 29–45.

[30] Kim JD, Kim CH, Kwon BS (2011) Regulation of mouse 4–1BB expression: multiple promoter usages and a splice variant. Mol Cells 31: 141–149. 21347708 10.1007/s10059-011-0018-6 PMC3932682

[31] Wilcox RA, Tamada K, Flies DB, Zhu G, Chapoval AI, et al (2004) Ligation of CD137 receptor prevents and reverses established anergy of CD8+ cytolytic T lymphocytes in vivo. Blood 103: 177–184. 12969968 10.1182/blood-2003-06-2184

[32] Kwon BS, Hurtado JC, Lee ZH, Kwack KB, Seo SK, et al (2002) Immune responses in 4-1BB (CD137)-deficient mice. J Immunol 168: 5483–5490. 12023342 10.4049/jimmunol.168.11.5483

