# Supplementary figures and images for: 4-1BB Signaling Breaks the Tolerance of Maternal CD8+ T Cells That Are Reactive with Alloantigens
Source: PLoS One. 2012 Sep 21;7(9):e45481. doi: 10.1371/journal.pone.0045481 (PMC3448654; doi:10.1371/journal.pone.0045481)

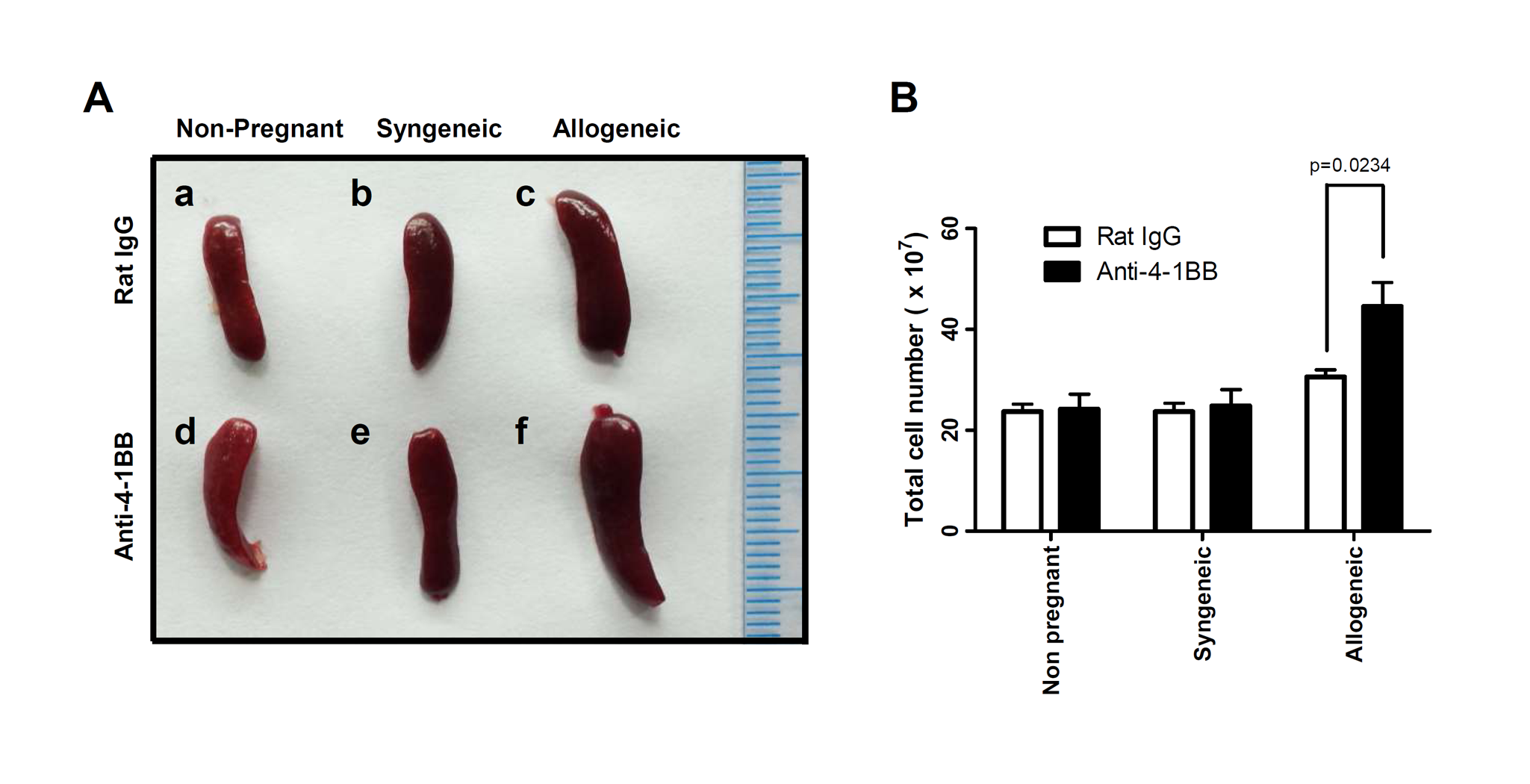

Supplement: Figure S1 — Impacts of 4-1BB triggering on splenocytes of the mice carrying syngeneic or allogeneic fetus. C57BL/6 female mice were time-mated with C57BL/6 (b, e) or DBA/2 (c, f) male mice. The mice with vaginal plug and non-pregnant C57BL/6 female mice were further injected i.p. with rat IgG or anti-4-1BB mAb at 8, 10, and 12 dpc as described above. The mice were sacrificed on 15 dpc and spleens were photographed (A) and total splenocytes were counted (B). (TIF) [file pone.0045481.s001.tif]
